# Covid-19 mortality is negatively associated with test number and government effectiveness

**DOI:** 10.1038/s41598-020-68862-x

**Published:** 2020-07-24

**Authors:** Li-Lin Liang, Ching-Hung Tseng, Hsiu J. Ho, Chun-Ying Wu

**Affiliations:** 1 Department of Business Management, National Sun Yat-sen University, No. 70, Lienhai Rd., Kaohsiung, 804 Taiwan; 2 Germark Biotechnology Co., Ltd., No. 21, Keyuan Rd., Taichung, 407 Taiwan; 3 Institute of Biomedical Informatics, National Yang-Ming University, No. 155, Sec. 2, Linong Street, Taipei, 112 Taiwan; 4 Institute of Biomedical Informatics, Institute of Public Health, Institute of Clinical Medicine, Faculty of Medicine, School of Medicine, National Yang-Ming University, No. 155, Sec. 2, Linong Street, Taipei, 112 Taiwan; 5 Division of Translational Research, Taipei Veterans General Hospital, No. 201, Sec. 2, Shipai Rd., Taipei, 112 Taiwan; 6 Department of Public Health, China Medical University, No. 91, Hsueh-Shih Rd., Taichung, 404 Taiwan

**Keywords:** Health care, Disease prevention, Preventive medicine

## Abstract

A question central to the Covid-19 pandemic is why the Covid-19 mortality rate varies so greatly across countries. This study aims to investigate factors associated with cross-country variation in Covid-19 mortality. Covid-19 mortality rate was calculated as number of deaths per 100 Covid-19 cases. To identify factors associated with Covid-19 mortality rate, linear regressions were applied to a cross-sectional dataset comprising 169 countries. We retrieved data from the Worldometer website, the Worldwide Governance Indicators, World Development Indicators, and Logistics Performance Indicators databases. Covid-19 mortality rate was negatively associated with Covid-19 test number per 100 people (RR = 0.92, *P* = 0.001), government effectiveness score (RR = 0.96, *P* = 0.017), and number of hospital beds (RR = 0.85, *P* < 0.001). Covid-19 mortality rate was positively associated with proportion of population aged 65 or older (RR = 1.12, *P* < 0.001) and transport infrastructure quality score (RR = 1.08, *P* = 0.002). Furthermore, the negative association between Covid-19 mortality and test number was stronger among low-income countries and countries with lower government effectiveness scores, younger populations and fewer hospital beds. Predicted mortality rates were highly associated with observed mortality rates (r = 0.77; *P* < 0.001). Increasing Covid-19 testing, improving government effectiveness and increasing hospital beds may have the potential to attenuate Covid-19 mortality.

## Introduction

Since the first report of the severe acute respiratory syndrome coronavirus 2 (SARS-CoV-2), causing coronavirus disease 2019 (Covid-19)^1,2^, more than 8.7 million people have been infected and more than 460 thousand have died worldwide as of June 20, 2020. The highly contagious Covid-19 has led to large numbers of infections, health care system overload, and lockdowns in many countries^3–5^.

A question central to the Covid-19 pandemic is why the Covid-19 mortality rate varies so greatly across countries, from over 16% in France and Belgium to less than 0.1% in Singapore and Qatar. Such wide variation implies that there are factors other than patient characteristics that determine Covid-19 mortality, such as government response. Patient-level studies have shown that Covid-19 mortality can be explained by age, obesity, and underlying diseases, such as hypertension, diabetes, and coronary heart disease, etc.^6–8^, as well as clinical symptoms, complications, hospital care, previous immunity and virus mutations^9,10^. These findings help health professionals to identify high-risk patients. However, this evidence alone may not be sufficient to support effective policies for reducing Covid-19 mortality.

This gap in Covid-19 research has been addressed by several studies. Some scholars have discussed the effectiveness of governments’ policies, such as quarantine or lockdown, in slowing the spread of Covid-19^3,11^. Others have suggested that projecting hospital utilization during the Covid-19 outbreak is necessary to assure the adequacy of resources to treat large numbers of patients^12^. A recent study analyzed the association between Covid-19 mortality and health care resource availability^13^. In addition, increasing Covid-19 testing has been advocated to attenuate its spreading^14^.

The resulting pieces of evidence have not been assembled or applied to explanations of country variations in Covid-19 mortalities. Countries vary widely in terms of capacities to prevent, detect and respond to disease outbreaks^15^. We aim to explore factors associated with Covid-19 mortalities at the country level. Specifically, we examined whether a key strategy, Covid-19 testing, can reduce Covid-19 mortalities. We also examined whether the severity of Covid-19 outbreak, as measured by the critical case rate and case number explains high numbers of Covid-19 mortalities. Furthermore, we investigated whether government effectiveness, or the government’s capacity to formulate and implement sound policies to tackle the crisis, can reduce Covid-19 mortality. Finally, this study analyzed the associations of Covid-19 mortality with proportions of aged persons, number of hospital beds, preexisting disease patterns and transport infrastructure, a proxy for human mobility.

## Methods

### Study design and data sources

For this worldwide cross-sectional study, we used data from open access databases. We retrieved Covid-19 related data from the website “Worldometer: coronavirus”^16^. This website has complied data from several important resources, such as the World Health Organization, U.S. Centers for Diseases Control and Prevention, and Computational Health Informatics Program of Harvard University. It has documented Covid-19 case numbers, death numbers, critical case numbers, and test numbers from more than 200 countries. We identified 7,732,952 Covid-19 cases with 428,248 deaths at 03:00 GMT on June 13, 2020 from the Worldometer database.

Government effectiveness information was retrieved from the Worldwide Governance Indicators (WGI) website^17^. WGI use perceptions-based data sources, covering over 200 countries and territories. Data sources of WGI include surveys of households and firms and expert assessments of various organizations^18^. Information regarding proportions of aged persons, hospital bed numbers, and disease patterns, was retrieved from the World Development Indicators (WDI)^19^. WDI are compiled by the World Bank and provide comprehensive cross-country comparable data on development. Data on quality of transport infrastructure was obtained from Logistics Performance Index (LPI) website^20^, which was based on surveys conducted by the World Bank in partnership with various institutions^21^. The most recent year for which WGI, WDI and LPI country data was available was 2018. After merging Covid-19 data with country-level data, the study sample consisted of totally 7,724,530 Covid-19 infected patients with 428,086 deaths in 169 countries. Countries were excluded from this analysis, if data for COVID-19 mortality rate was not available from public sources. The sample countries are described in Supplementary Table S1.

### Variables

Covid-19 mortality rate was defined as the number of deaths per 100 Covid-19 cases. Since the distribution of Covid-19 deaths was right skewed, we log-transformed the variable to make the data conform more closely to the normal distribution and to improve the model fit. The Covid-19 related factors were the test number per 100 people, case number per 1,000 people, and the critical case rate. The critical case rate was calculated by dividing the number of critical cases by the number of Covid-19 infected cases.

Government effectiveness was measured by WGI government effectiveness scores. These scores captured perceptions of a diverse group regarding the quality of public and civil services (e.g. education and basic health services), the quality of policy formulation and implementation, and the government commitment to such policies^18^. WGI applied a statistical method termed an unobserved component model to standardize data from various sources and to construct indicators. The scores for government effectiveness ranged from − 2.50 to 2.50, with a lower value indicating a lower level of effectiveness^18^. Population age structure was measured by the percentage of the population aged 65 or older. The number of beds was measured per 1,000 people. Disease patterns were measured by the percentage of all-cause deaths attributable to communicable diseases. The range of communicable diseases was all diseases excluding non-communicable diseases such as cancer and diabetes mellitus. Quality of transport infrastructure was measured by a LPI indicator, “quality of trade and transport-related infrastructure”. The indicator assessed the overall quality of ports, airports, rail, roads, and information technology. The quality score ranged from 1 (worst) to 5 (best), and was estimated to allow for cross-country comparisons^21^.

### Linear regression analyses

Simple linear regressions were first applied to investigate the correlation between Covid-19 mortality rate and test number, because the number of COVID-19 testing is more controllable by government than other predictors in our model. We ranked countries on the basis of their per capita incomes, government effectiveness scores, proportions of population aged 65 or older, and numbers of hospital beds. For each ranking, countries were divided into high, middle/moderate, and low. The goal was to examine whether the relationship between Covid-19 mortality and testing varied with country characteristics. Correlation coefficient and p-value of coefficient for test number were calculated for all subgroup analyses.

In the multiple regression analysis, Covid-19 mortality rate was regressed on Covid-19 test number, case number, critical case rate, government effectiveness score, proportion of population aged 65 or older, number of beds, deaths attributable to communicable diseases, and transport infrastructure quality score. Country populations were used as weights to account for unequal variances in the potential distribution of the disturbance term. The use of weights did not change regression results substantially. All analyses were performed using Stata 16 software (StataCorp Inc.).

### Validation study

The validity of our regression model was examined by comparing the observed Covid-19 mortality rates with the predicted mortality rates for individual countries. We drew a graph with observed and predicted mortality rates on the two axes. If the model fit well, we expected to see the data points scattered around the 45-degree cross line on the graph.

## Results

### Descriptive statistics

Table 1 summarizes the Covid-19 mortality rates and regression covariates. For the 169 studied countries, the mean Covid-19 mortality rate was 3.70% (95% CI 3.15 to 4.25%). The mean Covid-19 test number per 100 people was 3.75 (95% CI 2.82 to 4.69); the mean Covid-19 case number per 1,000 people was 1.69 (95% CI 1.20 to 2.18); and the mean critical case rate was 0.56% (95% CI 0.44 to 0.68). Moreover, the mean government effectiveness score was − 0.01 (95% CI − 0.17 to 0.16); the mean proportion of the population aged 65 or older was 9.17% (95% CI 8.15 to 10.18); the mean bed number per 1,000 people was 3.14 (95% CI 2.72 to 3.57); the mean communicable disease death rate was 31.04% (95% CI 27.50 to 34.58), and the mean transport infrastructure quality score was 2.75 (95% CI 2.64 to 2.86).

**Table 1:** Descriptive statistics of model variables.

	N	Mean	SE	95% CI
Covid-19 mortality rate (%)	169	3.70	0.28	3.15–4.25
**Covid-19 related factors**				
Test number per 100 people	153	3.75	0.47	2.82–4.69
Case number per 1,000 people	169	1.69	0.25	1.20–2.18
Critical case rate (%)^a^	120	0.56	0.06	0.44–0.68
**Country related factors**				
Government effectiveness score^b^	167	− 0.01	0.08	− 0.17–0.16
Population aged 65 or older (%)	162	9.17	0.51	8.15–10.18
Bed number per 1,000 people	146	3.14	0.22	2.72–3.57
Communicable disease death rate (%)	159	31.04	1.79	27.50–34.58
Transport infrastructure quality score^c^	153	2.75	0.05	2.64–2.86

^a^Critical case rate = number of critical cases/total number of cases.

^b^Range of data: from − 2.5 (worst) to 2.5 (best).

^c^Range of data: from 1 (worst) to 5 (best).

### Simple regression analyses: relationships between Covid-19 mortality rate and test number

**Figure 1:**
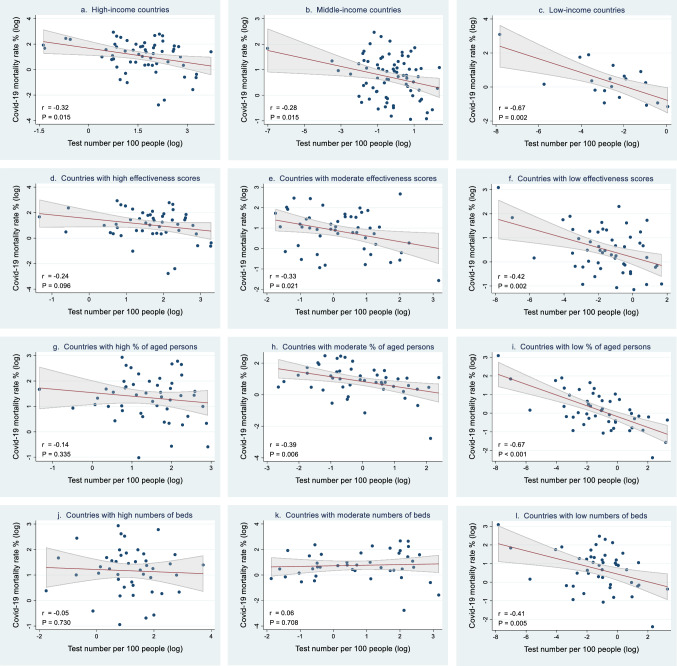
Correlation between Covid-19 mortality rate and test number. Countries were categorized by income group (**a**–**c**): (**a**) High-income (N = 59), (**b**) Middle-income (N = 75), (**c**) Low-income (N = 19); by governemnt effectiveness scores (**d**–**f**): (**d**) High effectivenss scores (N = 50), (**e**) Moderate effectiveness scores (N = 50), (**f**) Low effectiveness scores (N = 51); by percentage of people aged 65 or older (**g**–**i**): (**g**) High percentages of aged persons (N = 49), (**h**) Moderate percentages of aged persons (N = 49), (**i**) Low percentages of aged persons (N = 49); by number of hospital beds (**j**–**l**): (**j**) High numbers of beds (N = 45), (**k**) Moderate numbers of beds (N = 43), (**l**) Low numbers of beds (N = 46). Lines are linear predictions of Covid-19 mortality rate on test number. The 95% confidence intervals of the fitted values are shown by grey areas (r: correlation coefficient).

Relationships between Covid-19 mortality rate and test number are illustrated in Fig. 1. Figure 1a, 1b, and 1c demonstrates that Covid-19 mortality rate was negatively and significantly associated with test number for high-income (r = − 0.32, *P* = 0.015), middle-income (r = − 0.28, *P* = 0.015) and low-income (r = − 0.67, *P* = 0.002) countries, respectively. Figure 1e and 1f exhibits that the negative correlation between Covid-19 mortality rate and test number was significant for countries with moderate (r = -0.33, *P* = 0.021) and low (r = − 0.42, *P* = 0.002) government effectiveness scores, respectively. Figure 1h and 1i displays that the negative correlation was significant for countries with moderate (r = − 0.39, *P* = 0.006) and low (r = − 0.67, *P* < 0.001) percentage of aged persons, respectively. Finally, Fig. 1l reveals that the negative correlation was significant in countries with fewest beds (r = − 0.41, *P* = 0.005).

### Multiple regression analysis

Results of multiple regression for predicting Covid-19 mortality rates are shown in Table 2. Among the Covid-19 related factors, one additional Covid-19 screening test per 100 people was associated with a 8% reduction in mortality risk (RR = 0.92; 95% CI 0.87 to 0.96, *P* = 0.001). Among the country related factors, a 0.1 increase in government effectiveness score was associated with a 4% reduction in mortality risk (RR = 0.96; 95% CI 0.92 to 0.99, *P* = 0.017); a percentage point increase in the population aged 65 or older is associated with a 12% increase in mortality risk (RR = 1.12, 95% CI 1.07 to 1.17, *P* < 0.001). One additional bed per 1,000 people was associated with a 15% reduction in mortality risk (RR = 0.85; 95% CI 0.80 to 0.90, *P* < 0.001). A 0.1 increase in logistics infrastructure quality score was associated with a 8% increase in mortality risk (RR = 1.08; 95% CI 1.03 to 1.14, *P* = 0.002).

**Table 2:** Multiple regression for predicting Covid-19 mortality rates.

Predictors	RR^a^	SE^b^	P	95% CI
Test number per 100 people	0.92	0.02	0.001	0.87–0.96
Case number per 1,000 people	1.03	0.04	0.477	0.95–1.10
Critical case rate (%)	1.05	0.06	0.372	0.94–1.18
Government effectiveness score^c^	0.96	0.02	0.017	0.92–0.99
Population aged 65 or older (%)	1.12	0.02	< 0.001	1.07–1.17
Bed number per 1,000 people	0.85	0.03	< 0.001	0.80–0.90
Communicable disease death rate (%)	0.99	0.01	0.157	0.98–1.00
Transport infrastructure quality score^d^	1.08	0.03	0.002	1.03–1.14

A total of 101 countries were included in the regression analysis. The dependent variable was Covid-19 mortality rate % (log). The R-squared value was 0.58; adjusted R-squared value was 0.54.

^a^RR: relative risk. ^b^SE: standard errors. ^c,d^Both government effectiveness and infrastructure quality scores were multiplied by 10. Thus the corresponding relative risk should be interpreted on the basis of a 0.1 incremental increase in these indicators.

### Validation of the prediction model

**Figure 2:**
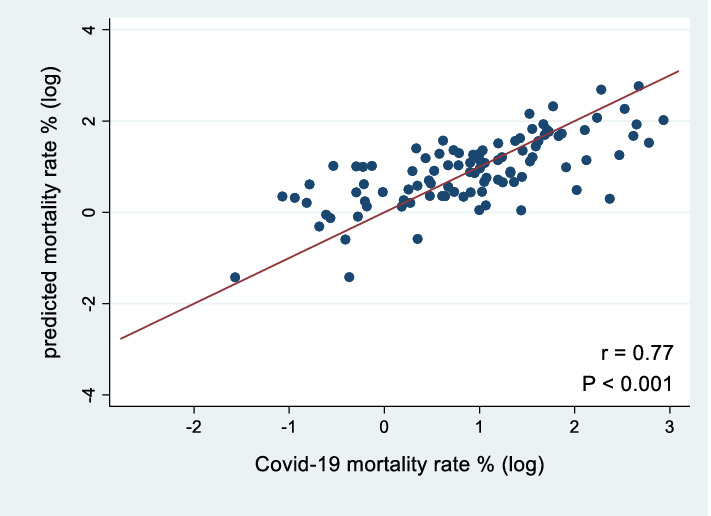
Correlation between observed and predicted Covid-19 mortality rates. The 45-degree line indicates equality of observed and predicted Covid-19 mortality rates (r: correlation coefficient; N = 99).

To validate our regression model, we examined the association between the predicted and the observed mortality rates for each country (Fig. 2). The predicted value was obtained from the multiple linear regression. The X axis was the observed morality rate and the Y axis was the predicted mortality rate. We excluded Singapore and Qatar from Fig. 2 because they were outliers. The predicted mortality rates were significantly and positively correlated with the observed mortality rates (r = 0.77, *P* < 0.001).

### Robustness analyses

As robustness checks, we included variables for GDP per capita, health expenditures and primary school enrolment rate in multiple regressions for Covid-19 mortality rate. The variables are summarized in Supplementary Table S2. None of the coefficients for these variables was statistically significant, and the main regression results did not change. Therefore, these variables were excluded from the final model. In addition, we conducted analyses for the relationships of Covid-19 mortality rate with GDP per capita and school enrolment rate for different income groups. The results are presented in Supplementary S3.

## Discussion

To the best of our knowledge, this is the first country level study to systematically examine the factors related to Covid-19 mortality. The multiple regression revealed that Covid-19 mortality rate is negatively associated with test number. The effectiveness of population screening for Covid-19 infection to reduce mortality risk is currently being debated. Those supporting screening suggest the beneficial effect of identifying asymptomatic patients to attenuate Covid-19 spread. Opponents argue that reduced mortality risk is mainly due to increased detection of asymptomatic patients. In the present study, we found that one additional test per 100 people was associated with a 8% reduction in mortality rate, even after adjusting for case number, critical case rate, and various country-related factors.

Notably, simple regression analyses indicated that the negative association of Covid-19 mortality with test number varied with country characteristics. Low-income countries and countries which had the lowest government effectiveness scores, lowest proportions of aged persons, and fewest beds (i.e. those at the bottom one-third of ranking) exhibited the most negative correlation (in terms of correlation coefficient) between Covid-19 mortality and testing. We re-examined these results by including interactions terms between test number and country characteristics; similar conclusions were reached. These results suggest that scaling up testing might potentially serve as an effective approach to attenuate mortality when governments were less effective in controlling disease outbreaks or when hospital beds were less sufficient.

Greater government effectiveness was found in this study to be associated with lower Covid-19 mortality rates. This indicator captures capacity of government to effectively formulate and implement sound policies, and is a key dimension of good governance. Good governance is essential to long-term development outcomes, such as per capita incomes^22^. The present study demonstrated that for short-term crises such as the Covid-19 outbreak, government effectiveness remains critical. For example, an effective government would respond to Covid-19 pandemic proactively by making policies to ensure sufficient supply of personal protective equipment^23^. Quick implementation of effective quarantine, lockdown and screening policies^3,24,25^, as well as provision of good public health services in managing and treating Covid-19 patients, also require an effective government^26^.

Recent Covid-19 clinical studies have reported associations for mortality with old age and multiple comorbidities^6,7,27^. We confirmed these observations. Countries with higher proportions of people aged 65 or older had significantly higher mortality rates (*P* < 0.001). In the present study, bed number was negatively and significantly associated with Covid-19 mortality rate (*P* < 0.001). This finding supports the argument that hospital bed is a critical input in treating Covid-19 infected patients who need intensive care^5^. In addition, countries with better trade and transport-related infrastructure appeared to have higher Covid-19 mortality rates (*P* = 0.002). A possible explanation is that transport infrastructure facilitated human mobility and movement of goods, which might increase transmissions of Covid-19 among high-risk populations.

There are several limitations to the present study. First, this study is based on Covid-19 cases reported by countries. Inaccurate reporting and the rapid increases in cases may have influenced the predictive power of our model. However, the trends in the prognostic factors for predicting mortality rates may not have changed. Second, the lack of completeness of the database limits our analyses in certain countries, for example test numbers in China and critical case numbers in New Zealand and Indonesia. Third, the Covid-19 related factors used in the present study are from country-level data, not patient-level data. If worldwide patient-level data is made available for analyses, the prediction accuracy will further improve. Fourth, we selected only a limited number of factors that potentially determine the Covid-19 mortality in a country. Future studies may explore other country-related factors to improve the prediction accuracy. Finally, acquired community immunity after the worldwide spread of Covid-19 may change the prediction accuracy. However, the results of this study can still contribute to future pandemic-related policymaking at the country level.

In conclusion, we found that higher Covid-19 mortality is associated with lower test number, lower government effectiveness, aging population, fewer beds, and better transport infrastructure. Increasing Covid-19 test number and improving government effectiveness have the potential to reduce Covid-19 related mortality.

## Supplementary information

Supplementary information.
