# Supplementary material for: Covid-19 mortality is negatively associated with test number and government effectiveness
Source: Sci Rep. 2020 Jul 24;10:12567. doi: 10.1038/s41598-020-68862-x (PMC7381657; doi:10.1038/s41598-020-68862-x)
Supplement: Supplementary file 1 — Supplementary information. [file 41598_2020_68862_MOESM1_ESM.pdf]

Covid-19 mortality is negatively associated with test number and government effectiveness

Short title: Factors associated with Covid-19 mortality

Li-Lin Liang, Ph.D[+].[1], Ching-Hung Tseng, Ph.D. [2], Hsiu J. Ho, Ph.D.[3], and Chun-Ying Wu[+]*, M.D., Ph.D.[4-6]
.

[1]Department of Business Management, National Sun Yat-sen University, Kaohsiung, Taiwan

[2]Germark Biotechnology Co., Ltd., Taichung, Taiwan

[3]Institute of Biomedical Informatics, National Yang-Ming University, Taipei, Taiwan

[4]Institute of Biomedical Informatics, Institute of Public Health, Institute of Clinical Medicine, Faculty of Medicine, National Yang-Ming University, Taipei, Taiwan

[5]Division of Translational Research, Taipei Veterans General Hospital, Taipei, Taiwan

[6]Department of Public Health, China Medical University, Taichung, Taiwan

[+] The two authors contributed equally to this work

* Correspondence:

Chun-Ying Wu, M.D., MPH, Ph.D.
Institute of Biomedical Informatics, School of Medicine, National Yang-Ming University
No. 155, Section 2, Linong Street, Taipei 11221, Taiwan
E-mail: cywu4@ym.edu.tw
Tel: +886-2-28267155;
Fax: +886-2-28202508

SUPPLEMENTARY MATERIAL

S1. Sample countries

Our study sample consisted of 169 countries for which data for Covid-19 case number and death number were publicly available. Supplementary Table S1 lists sample countries and their corresponding Covid-19 mortality rates and income levels. According to World Bank classification (Atlas method), a country was classified as low-income if its per capita gross national income in 2018 was no more than USD 1,025, as middle-income if it was more than USD 1,025 and no more than USD 12,375, and as high-income if it was more than USD 12,375. On the basis of the 2018 criterion, 59 out of 169 countries (35%) were classified as high-income, 82 were middle-income (48%), and 28 were low-income (17%).

Table S1 Countries in the study sample

Country	Covid-19 mortality (%)	Income level	Country	Covid-19 mortality (%)	Income level
Afghanistan	1.89	L	Kazakhstan	0.50	M
Albania	2.54	M	Kenya	2.90	M
Algeria	7.02	M	Korea, Rep.	2.30	H
Andorra	5.98	H	Kuwait	0.82	H
Angola	3.85	M	Kyrgyz Republic	1.20	M
Antigua and Barbuda	11.54	H	Latvia	2.46	H
Argentina	2.73	M	Lebanon	2.18	M
Armenia	1.69	M	Liberia	7.60	L
Aruba	2.97	H	Libya	1.47	M
Australia	1.40	H	Liechtenstein	1.22	H
Austria	3.96	H	Lithuania	4.21	H
Azerbaijan	1.23	M	Luxembourg	2.71	H
Bahamas, The	10.68	H	Madagascar	0.81	L
Bahrain	0.21	H	Malawi	0.83	L
Bangladesh	1.34	M	Malaysia	1.42	M
Barbados	7.29	H	Maldives	0.40	M
Belarus	0.57	M	Mali	5.76	L
Belgium	16.13	H	Malta	1.40	H
Belize	10.00	M	Marshall Islands	6.93	M
Benin	1.29	L	Mauritania	5.15	M
Bermuda	6.38	H	Mauritius	2.97	M
Bolivia	3.30	M	Mexico	11.82	M

Bosnia and Herzegovina	5.63	M	Moldova	3.47	M
Botswana	2.08	M	Monaco	4.04	H
Brazil	5.05	M	Montenegro	2.78	M
Brunei Darussalam	1.42	H	Morocco	2.46	M
Bulgaria	5.39	M	Mozambique	0.39	L
Burkina Faso	5.94	L	Myanmar	2.30	M
Burundi	1.18	L	Nepal	0.32	L
Cabo Verde	0.86	M	Netherlands	12.49	H
Cameroon	2.44	M	New Zealand	1.46	H
Canada	8.22	H	Nicaragua	3.76	M
Cayman Islands	0.53	H	Niger	6.65	L
Central African Republic	0.34	L	Nigeria	2.63	M
Chad	8.49	L	North Macedonia	4.62	M
Chile	1.78	H	Norway	2.81	H
China	5.58	M	Oman	0.46	H
Colombia	3.30	M	Pakistan	1.96	M
Comoros	1.23	M	Panama	2.19	H
Congo, Dem. Rep.	2.18	L	Paraguay	0.88	M
Congo, Rep.	3.30	M	Peru	2.86	M
Costa Rica	0.74	M	Philippines	4.24	M
Cote d'Ivoire	0.96	M	Poland	4.28	H
Croatia	4.76	H	Portugal	4.16	H
Cuba	3.76	M	Qatar	0.09	H
Cyprus	1.84	H	Romania	6.45	M
Czech Republic	3.31	H	Russian Federation	1.31	M
Denmark	4.91	H	Rwanda	0.39	L
Djibouti	0.86	M	San Marino	6.05	H
Dominican Republic	2.58	M	Sao Tome & Principe	1.88	M
Ecuador	8.36	M	Saudi Arabia	0.74	H
Egypt, Arab Rep.	3.44	M	Senegal	1.15	M
El Salvador	1.95	M	Serbia	2.07	M
Equatorial Guinea	0.92	M	Sierra Leone	4.62	L
Estonia	3.50	H	Singapore	0.06	H
Eswatini	0.64	M	Slovak Republic	1.82	H
Ethiopia	1.61	L	Slovenia	7.32	H
Finland	4.59	H	Somalia	3.38	L
France	18.79	H	South Africa	2.19	M
Gabon	0.66	M	South Sudan	1.44	L

Gambia, The	3.57	L	Spain	9.35	H
Georgia	1.54	M	Sri Lanka	0.59	M
Germany	4.73	H	Sudan	6.29	M
Ghana	0.44	M	Suriname	1.60	M
Greece	5.89	H	Sweden	9.77	H
Guatemala	3.91	M	Switzerland	6.24	H
Guinea	0.54	L	Syrian Arab Republic	3.66	L
Guinea-Bissau	1.03	L	Tajikistan	1.00	L
Guyana	7.55	M	Tanzania	4.13	L
Haiti	1.62	L	Thailand	1.85	M
Honduras	3.76	M	Togo	2.48	L
Hong Kong, China	0.36	H	Trinidad and Tobago	6.84	H
Hungary	13.69	H	Tunisia	4.48	M
Iceland	0.55	H	Turkey	2.73	M
India	2.87	M	Ukraine	2.92	M
Indonesia	5.63	M	United Arab Emirates	0.69	H
Iran, Islamic Rep.	4.74	M	United Kingdom	14.16	H
Iraq	2.79	M	United States	5.52	H
Ireland	6.75	H	Uruguay	2.72	H
Israel	1.60	H	Uzbekistan	0.39	M
Italy	14.48	H	Venezuela, RB	0.80	M
Jamaica	1.63	M	West Bank and Gaza	0.61	M
Japan	5.32	H	Yemen, Rep.	21.99	L
Jordan	0.98	M	Zambia	0.76	M
			Zimbabwe	1.17	M

Notes: H=High-income country; M=Middle-income country; L=Low-income countries. Data for Covid-19 mortality rate were collected from the Worldometer website <https://www.worldometers.info/coronavirus/#countries> (access date: June 13, 2020).

S2. Robustness analyses: variables excluded from the Covid-19 mortality model

This appendix presents variables that potentially explained cross-country variation in Covid-19 mortality rates, but were not included in the final model. The excluded variables were those related to economic development, health care expenditures, and education. The data source was World Development Indicators described in the main text. Our conjecture was that wealthier countries or countries traditionally spent more on health care might have more resources to tackle Covid-19. Countries where people had better education may have better health literacy, which may increase compliance with government control measures. These variables were added one at a time to our multiple linear regression model. None of the coefficients for these variables was statistically significant, and adding these variables did not increase the explanatory power of the model. Thus they were excluded from our final model.

Table S2 Variables analyzed but not included in the final model

Variables
**Economic development**
GDP per capita, PPP (current international $)
GDP per capita growth (annual %)
**Health care expenditures**
Current health expenditure per capita, PPP (current international $)
Current health expenditure (% of GDP)
Domestic general government health expenditure (% of general government expenditure) (a proxy for prioritization of health)
**Educational level**
Primary school enrolment rate (% gross)

Note: GDP: Gross Domestic Product. PPP: Purchasing Power Parity. The year of data was 2018, except for primary school enrolment rate, for which data was for year 2017. The data source was World Development Indicators database.

S3. Relationships of Covid-19 mortality with GDP per capita and school enrolment rate, by income groups

Following Supplementary S2, this appendix further investigated in different income groups, the relationship of Covid-19 mortality rate with economic development and education level. Economic development was measured by GDP per capita (PPP, current international $). Educational level was measure by the primary school enrolment rate (% gross). The results from simple linear regressions were illustrated in Figures S3.1 and S3.2.

Figure S3.1 shows that Covid-19 mortality rate was negatively and significantly associated with per capita GDP only for high-income countries (r=-0.42, P=0.001). Figure S3.2 shows that Covid-19 mortality was negatively and significantly associated with primary school enrolment rate only for low-income countries (r=-0.75, P<0.001).

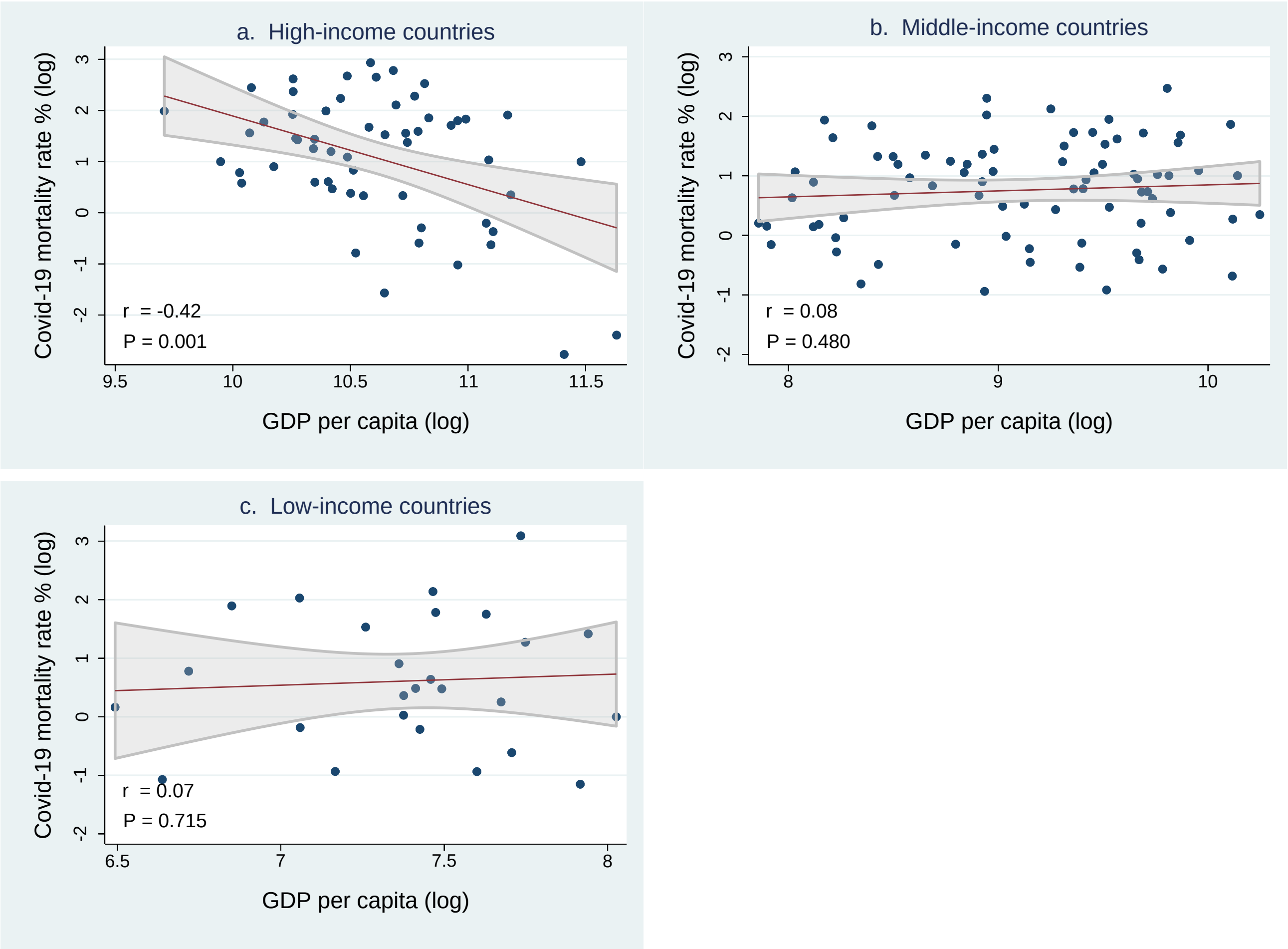

Figure S3.1 Correction between Covid-19 mortality rate and GDP per capita, by income level: a. High-income countries (N=56), b. Middle-income countries (N=81), c. Low-income countries (N=26). Red lines are linear predictions of Covid-19 mortality rates on GDP per capita. The 95% confidence intervals of the fitted values are shown by grey areas (r = correlation coefficient).

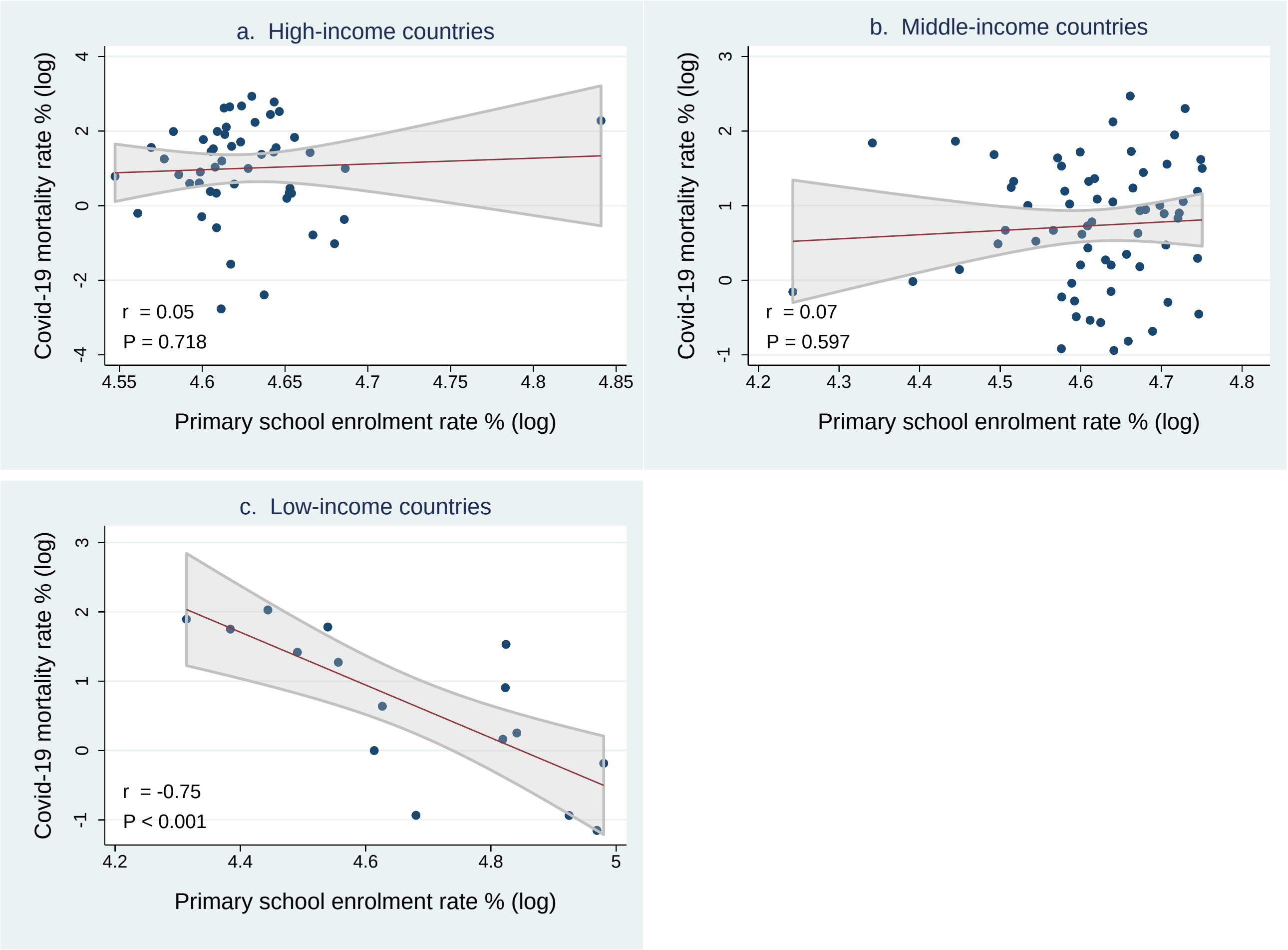

Figure S3.2 Correlation between Covid-19 mortality rate and primary school enrolment rate, by income level: a. High-income countries (N=50), b. Middle-income countries (N=65), c. Low-income countries (N=16). Red lines are linear predictions of Covid-19 mortality rates on primary school enrolment rates. The 95% confidence intervals of the fitted values are shown by grey areas (r = correlation coefficient).
